# Extracorporeal membrane oxygenation offers long-term survival in childhood leukemia and acute respiratory failure

**DOI:** 10.1186/s13054-018-2134-6

**Published:** 2018-09-22

**Authors:** Gerard Cortina, Nikolaus Neu, Gabriele Kropshofer, Bernhard Meister, Uwe Klingkowski, Roman Crazzolara

**Affiliations:** Department of Pediatrics, Medical University of Innsbruck, Anichstrasse 35, A-6020 Innsbruck, Austria

**Keywords:** Extracorporeal membrane oxygenation, Childhood leukemia, Acute respiratory failure

Dear Editor:

Survival for childhood cancer has dramatically improved, particularly for acute lymphoblastic leukemia, reaching over 90% overall survival in industrialized countries [1]. However, some patients may encounter severe adverse events, limiting this high success rate. ARF is one of the most serious complications and is associated with high mortality if conventional therapy fails [2]. Escalation to ECMO has rarely been used in patients with malignancy due to its limited success rates and higher risk for infectious and bleeding complications [3–5].

We report on a single centre experience of ECMO on patients with childhood leukemia and ARF. This retrospective study was approved by the local research ethics committee. Nine patients with childhood leukemia received ECMO in induction treatment (8/9 at first remission, 1/9 at second remission) between January 2004 and June 2017. Details on these patients are provided in Table 1. ARF resulted from pulmonary infections (two patients with *Candida albicans*, one patient with *Aspergillus terreus*, four patients with no organisms identified) and pulmonary non-infectious complications (one patient with transfusion-related acute lung injury and one patient with leukemic infiltration). Median duration of mechanical ventilation before ECMO was 3 days (range 0.4–14). The median duration of ECMO support was 14 days (range 2–24). Five (56%) patients survived ECMO und four (44%) survived to hospital discharge. When compared to survivors, non-survivors had a significantly higher vasoactive inotrope score (VIS) at ECMO initiation (85 vs. 11; *p* = 0.032), including two patients requiring veno-arterial cannulation. Time on ECMO support was shorter (5 vs. 15 days; *p* = 0.032) in non-survivors and was stopped because of multiorgan failure (22%), intracranial bleeding (11%) and progressive leukemia (11%). One patient (11%) recovered from hematopoietic stem cell transplantation performed on ECMO, but died two months later of septic shock. Moreover, non-survivors had significantly lower platelet count on ECMO (30 ×  10^3^/μL vs 98 × 10^3^/μL; *p* = 0.041). Eight (89%) patients received chemotherapy in the four weeks prior to and five (56%) were neutropenic at ECMO cannulation. Neutropenic patients did not have higher mortality compared to those without neutropenia (3/5 vs 2/4).

**Table 1:** Clinical characteristics and demographics of patients on ECMO

Clinical characteristics	N	Demographics	All	Survivors	Non-survivors	*p*-value
Diagnosis		Age (years)	14 (1–18)	9 (4–16)	16 (1–18)	0.286
ALL	5	Weight (kg)	47 (7–74)	26 (12–50)	56 (7–74)	0.286
AML	3	Pre ECMO				
JMML	1	pH	7.2 (7.0–7.6)	7.3 (7.0–7.6)	7.2 (7.0–7.4)	0.413
Reason for ARF		Lactate (mg/dL)	17 (7–68)	17 (7–24)	17 (8–68)	0.556
Fungal infection	3	pO2/FiO2	47 (32–67)	66 (32–67)	44 (34–50)	0.286
Pulmonary infection^a^	4	VIS score	45 (5–160)	11 (5–45)	85 (22–160)	0.032
TRALI	1	Platelet count (× 10^3^/μL)	27 (14–214)	145 (26–214)	27 (14–53)	0.111
Leukemic infiltration	1	Ventilation days	3 (0.4–14)	4.5 (1–13)	2 (0.4–12)	0.556
Causes of death on ECMO		During ECMO				
Intracranial hemorrhage	1	Platelet count (× 10^3^/μL)	35 (19–106)	98 (22–106)	30 (19–48)	0.041
Multiorgan failure	2	Platelets transfusions / day	2.2 (0.2–3.8)	0.5 (0.2–2.2)	3.3 (1.7–4.7)	0.111
Leukemic infiltration	1	VV Cannulation	7	5	2	
Outcome on ECMO		VA Cannulation	2	0	2	
Survived on ECMO	5	Major bleeding	4	0	4	
Discharged from hospital	4	Need for CRRT	3	0	3	
Survived long-term	4	ECMO Duration (days)	14 (2–24)	15 (9–24)	5 (2–17)	0.032

^a^no organism detected

*ALL* acute lymphoblastic leukemia, *AML* acute myeloid leukemia, *JMML* juvenile myelomonocytic leukemia, *ARF* acute respiratory failure, ^a^no organism detected, *ECMO* extracorporeal membrane oxygenation, *TRALI* transfusion-related acute lung injury, *VIS* vasoactive inotrope score = dose of dopamine (μg/kg/min) + dose of dobutamine (μg/kg/min) + 100 x dose of adrenaline (μg/kg/min) + 100 x dose of noradrenaline (μg/kg/min) + 10 x milrinone dose (μg/kg/min) + 10,000 x dose of vasopressin (U/kg/min), *VV* veno-venous, *VA* veno-arterial, *CRRT* continuous renal replacement therapy

All four survivors are in complete oncologic remission at a median follow-up of 8.4 years (range 1.8–13.1), are restored to full health, and are all engaged to full-time study or work. Our data is limited by a small sample size and by its retrospective analysis. Nevertheless, it indicates that ECMO provides an effective rescue therapy in childhood leukemic patients with ARF.

## Abbreviations

ARF: Acute respiratory failure
ECMO: Extracorporeal membrane oxygenation
